# PReS-FINAL-2015: Demographic, clinical and laboratory features of juvenile dermatomyositis in Croatia: retrospective study over the last 22 years

**DOI:** 10.1186/1546-0096-11-S2-P28

**Published:** 2013-12-05

**Authors:** M Frković, I Malčić, I Rukavina, V Rožmanić, K Markičević, N Turjak, M Jelušić

**Affiliations:** 1 University Hospital Centre Zagreb, Zagreb, Croatia; 2 University Hospital Centre Rijeka, Rijeka, Croatia; 3 University Hospital Centre "Sisters of Mercy", Zagreb, Croatia; 4 University Hospital Centre Split, Split, Croatia

## Introduction

Juvenile dermatomyositis (JDM) is the most common among the idiopathic inflammatory myopathies that occur during the childhood.

## Objectives

To analyze the disease characteristics, treatment modalities and outcome of juvenile dermatomyositis (JDM) in Croatian children.

## Methods

We reviewed medical records of all patients aged ≤ 18 years at disease onset who were diagnosed with JDM during the period 1990-2012 in the pediatric departments of four university-affiliated tertiary care hospitals in the three largest cities in Croatia: Zagreb, Rijeka, and Split.

## Results

JDM was diagnosed in 32 patients (16 girls and 16 boys) with mean age at the disease onset 8.8 years (boys: 7.5 years, girls: 10.2 years). Average period between disease onset and diagnosis was 9.8 months. Major clinical signs at the first visit were muscle weakness and/or pain (78% patients) and heliotrope rash (65% patients). Diagnosis was confirmed with laboratory findings (avarage CK values at the begining of treatment were 1571 ku/L and avarage LDH values were 735.5 ku/L), postive EMNG (performed in 75% patients), muscle biopsy (performed in 68% patients) and thigh muscles MR findings (performed in 12.5% patients).

Seven patients (21.8%) had severe form of JDM with vasculopathy: two with acute gastrointestinal perforation and bleeding, one with urinary tract involvement and chronical hematuria and four with multysistem involvement - brain oedema with hallucinations, respiratory distress syndome, myocarditis, gastrointesinal bleeding, one of them also with retinal cotton-wool spots and papillar oedema.

In all cases therapy included corticosteroids, in most cases combined with methotrexate and IV imunoglobulines, in cases with severe vasculopathy also combined with cyclophosphamide and plasmaphereis. In two patients with poor response to standard multiple drug therapy, anti-TNF therapy (infliximab) was introduced: in 9 years old girl during acute phase of disease characterised with emphasised vasculopathy and in 16 years boy with prolonged, chronical course of disease characterised with extensive, progressive calcionosis.

Three patients died (9.3%): two during the acute phase of disease because of gastrointestinal perforation, one during the follow up because of cardiac decompensation. In four patients (12.5%) disease assumed chronic course with calcinosis, despite all applied therapy.

In a girl with severe acute multysistem involvement including retinal cotton-wool spots and papillar oedema, early introduction of infliximab resulted in rapid, complete resolution of eye changes and excellent, fast general recovery. In a boy with chronical course of diasease characterised with progressive, extensive calcionsis, infliximab stopped further progression of disease.

## Conclusion

Early diagnosis as well as early agressive therapy (including anti-TNF) were the key of favourable outcomes in most patients enrolled in our study. In this respect, to our opinion, anti-TNF therapy should be considered as a part of an early treatment, especially in cases with severe, progressive forms of JDM.

## Disclosure of interest

None declared.

